# The Tumor Suppressor Par-4 Regulates Adipogenesis by Transcriptional Repression of PPARγ

**DOI:** 10.3390/cells13171495

**Published:** 2024-09-05

**Authors:** James Sledziona, Ravshan Burikhanov, Nathalia Araujo, Jieyun Jiang, Nikhil Hebbar, Vivek M. Rangnekar

**Affiliations:** 1 Department of Toxicology and Cancer Biology, University of Kentucky, 538 Healthy Kentucky Research Building, 760 Press Avenue, Lexington, KY 40536, USA; james.sledziona@osumc.edu (J.S.); n.vitoria.araujo@gmail.com (N.A.); nikhilhebbar@gmail.com (N.H.); 2 The Ohio State University James Comprehensive Cancer Center, The Ohio State University, Columbus, OH 43210, USA; 3 Radiation Medicine, University of Kentucky, Lexington, KY 40536, USA; rburi2@uky.edu (R.B.); jieyun.jiang2@uky.edu (J.J.); 4 Microbiology, Immunology and Molecular Genetics, University of Kentucky, Lexington, KY 40536, USA; 5 Markey Cancer Center, University of Kentucky, Lexington, KY 40536, USA

**Keywords:** Par-4, adipogenesis, PPARγ, tumor suppressor

## Abstract

Prostate apoptosis response-4 (Par-4, also known as PAWR) is a ubiquitously expressed tumor suppressor protein that induces apoptosis selectively in cancer cells, while leaving normal cells unaffected. Our previous studies indicated that genetic loss of Par-4 promoted hepatic steatosis, adiposity, and insulin-resistance in chow-fed mice. Moreover, low plasma levels of Par-4 are associated with obesity in human subjects. The mechanisms underlying obesity in rodents and humans are multi-faceted, and those associated with adipogenesis can be functionally resolved in cell cultures. We therefore used pluripotent mouse embryonic fibroblasts (MEFs) or preadipocyte cell lines responsive to adipocyte differentiation cues to determine the potential role of Par-4 in adipocytes. We report that pluripotent MEFs from Par-4^−/−^ mice underwent rapid differentiation to mature adipocytes with an increase in lipid droplet accumulation relative to MEFs from Par-4^+/+^ mice. Knockdown of Par-4 in 3T3-L1 pre-adipocyte cultures by RNA-interference induced rapid differentiation to mature adipocytes. Interestingly, basal expression of PPARγ, a master regulator of de novo lipid synthesis and adipogenesis, was induced during adipogenesis in the cell lines, and PPARγ induction and adipogenesis caused by Par-4 loss was reversed by replenishment of Par-4. Mechanistically, Par-4 downregulates PPARγ expression by directly binding to its upstream promoter, as judged by chromatin immunoprecipitation and luciferase-reporter studies. Thus, Par-4 transcriptionally suppresses the PPARγ promoter to regulate adipogenesis.

## 1. Introduction

Prostate apoptosis response-4 (Par-4) is a ubiquitously expressed tumor suppressor protein present in a wide range of vertebrates [1,2]. Loss of Par-4 expression via mechanisms such as methylation-dependent downregulation of the Par-4 promoter, inactivation of Par-4 protein by AKT-mediated phosphorylation, or spontaneous mutation, has been associated with several forms of human cancer [3,4,5,6]. Genetic knockout of Par-4 in mice results in spontaneous tumors, as well as hormone-or chemical-inducible tumors in multiple tissues [7]. Several studies have demonstrated that reduced Par-4 expression and activity correlates with unfavorable chemotherapeutic treatment prognosis and reduced radiosensitivity of breast and colon tumors [8,9,10]. On the other hand, overexpression of Par-4 induces apoptosis in cancer cell lines and inhibits tumor growth in mice, while leaving normal cells unaffected [1,11,12]. Accordingly, immunocompetent mice overexpressing Par-4 or its central core domain SAC (selective for apoptosis in cancer), which is necessary and sufficient for apoptosis in cancer cells, exhibit a normal lifespan and are resistant to the growth of spontaneous and oncogene-inducible tumors [11,12].

Par-4 is localized to various intracellular compartments, such as the cytoplasm, endoplasmic reticulum (ER), and nucleus, and is also secreted [1,13]. The carboxyl terminus of Par-4 contains a leucine zipper (LZ) domain that promotes Par-4 binding with partner proteins to regulate apoptosis [1]. The SAC domain also contains a nuclear localization sequence (NLS-designated: NLS2) that permits entry of intracellular Par-4 into the nucleus to inhibit NF-κB transcription activity and induce apoptosis in cancer cells [1]. Incidentally, another NLS domain (NLS1) is present near the N-terminus, but it is dispensable for Par-4 nuclear entry [14]. Par-4 has been shown to function as a transcriptional corepressor in reporter assays, and the prosurvival gene *Bcl2* is transcriptionally corepressed by Par-4 via interaction of its LZ domain with the WT1 protein in the nucleus [15,16]. Moreover, the Par-4/THAP1 complex and Notch3 competitively bind to the CCAR1 promoter and modulate alternative pre-mRNA splicing of CCAR1 [17].

Cellular metabolism can be modulated by altered expression or activation of oncogenes such as Ras and Myc, or tumor suppressors such as p53 and Par-4 [18,19,20,21]. These observations extend to obesity or adipogenesis, and a pertinent example is the tumor suppressor p53 that can regulate the transcription of genes associated with lipogenic enzymes [22,23,24]. Interestingly, genetic loss of Par-4 in mice induces p53 expression that regulates obesity via induction of complement factor C3 and its breakdown product acyl-stimulating protein, as well as by modulating lipoprotein lipase (LPL) in adipose tissues [21].

Peroxisome proliferator-associated receptor gamma (PPARγ) is a nuclear hormone receptor and transcription factor that is responsible for modulating transcription of multiple genes related to insulin response, lipid transport, metabolism, and adipogenesis. PPARγ is considered the adipogenic master regulator, and mutations in this gene promote lipodystrophy and hyperinsulinemia [25]. PPARγ occurs primarily as two isoforms, PPARγ1 and PPARγ2 [26]. These two isoforms, encoded by a single gene, begin transcription at different promoter sites [26] and exhibit disparate tissue distribution. PPARγ1 is widely distributed with a low level of expression, particularly concentrated in muscle and liver, and PPARγ2, by contrast, is predominantly expressed in adipose tissue where it is involved in adipocyte formation [27]. PPARγ2 is also expressed in liver hepatocytes and regulates SREBP-1 and FASN to induce de novo fatty acid synthesis [28,29]. Excess fat synthesized in the liver or from food is stored in adipose tissue. Adipose tissue contributes little by way of de novo lipid synthesis of fatty acids that are stored as triglyceride in lipid droplets. On the other hand, pre-adipocytes that can differentiate into mature adipocytes in cell culture almost exclusively rely on de novo lipogenesis for fatty acid synthesis and formation of lipid droplets, because the fat content of the cell culture medium is very low. Interestingly, PPARγ2 also regulates the process of adipogenesis, primarily inducing the maturation of pre-adipocytes to adipocytes [30].

Given our recent findings on obesity in Par-4 knockout mice [21], we determined whether Par-4 depletion would potentiate adipogenic reprogramming in undifferentiated fibroblasts. We used well-established cell culture models of adipogenesis to determine whether downregulation of Par-4 is a requisite for differentiation of adipocytes from progenitor cells. Our results suggest that Par-4 regulates the maturation of adipocytes and lipid accumulation through transcriptional repression of PPARγ2, a master regulator of adipogenesis.

## 2. Materials and Methods

### 2.1. Animals

C57BL/6 mice were purchased from The Jackson Laboratory (Bar Harbor, ME, USA) to prepare wild-type control MEFs. Par-4^fl/fl^ mice, generated on the C57BL/6 background by Taconic Biosciences (Germantown, NY, USA), were described previously by us [21]. These mice contain loxP sites flanking the second exon of Par-4. These animals were crossed with a ROSA-26-Cre mouse and, after genotyping the resulting offspring, the Par-4^+/−^ heterozygous mice were crossed to generate offspring with exon 2 of Par-4 excised in both alleles (designated Par-4^−/−^) [21]. Western blot analysis of various tissues and MEFs were used to verify Par-4 expression in these mice [21]. The experiments previously performed on these mice [21] were approved by the Institutional Animal Care and Use Committee of the University of Kentucky.

### 2.2. Cell Culture

MEFs were prepared from Par-4^−/−^ mice and Par-4^+/+^ wild-type littermate control embryos [21]. MEFs were maintained in Dulbecco’s Modified Eagle’s Medium (DMEM) (Mediatech, Inc.; Manassas, VA, USA), supplemented with 10% fetal bovine serum (DMEM+10% FBS), (Sigma Aldrich; St. Louis, MO, USA). Mouse NIH 3T3 and 3T3-L1 cells were obtained from ATCC and stocks were maintained in DMEM+10% FBS. Human breast cancer cells MCF7 with CRISPR/Cas9 knockout of Par-4/PAWR (MCF7 Par-4 KO) and MCF7 control cells were purchased from Synthego Corporation (Redwood City, CA, USA) and cultured in RPMI (Mediatech, Inc.; Manassas, VA, USA) +10% FBS.

### 2.3. Constructs and RNA-Interference

Adenoviral constructs for expression of Par-4, GFP-Par-4, and GFP have been previously described [1,14]. Par-4 and PPARγ siRNAs, as well as control siRNAs (siRNA A and siRNA B) were obtained from Santa Cruz Biotechnology (SCBT; Dallas, TX, USA).

Transfection with siRNA duplexes was performed in six-well plates according to manufacturer instructions. Briefly, the cells were plated 24 h prior to transfections, at a density of 10^6^ cells/well. At time of transfection for each well, siRNA duplexes (1 μg) were combined with DharmaFECT 1 transfection reagent (GE Life Sciences, Marlborough, MA, USA) in a volume of 200 µL serum and antibiotic-free DMEM and allowed to complex for 25 min. The complexes were added to each well in serum-free DMEM and permitted to transfect for five hours prior to replacing the medium with 10% FBS-supplemented DMEM. Cells transiently transfected with siRNA were used for experiments 48–72 h post-transfection. Cells containing shRNA were selected in medium containing puromycin (3 μg/mL). The cells were verified for knockdown by Western blot analysis.

### 2.4. In Vitro Adipogenesis

Early passage (<10 passages) MEF cells were allowed to grow for two days post-confluence in DMEM+10% FBS, which was their normal growth medium (control medium). After this point, the growth medium was replaced with adipocyte differentiation medium; i.e., DMEM+10% FBS, further supplemented with 10 µg/mL bovine insulin, 10 µM troglitazone, 500 µM 3-isobutyl-1-methylxanthine (IBMX), and 1 µM dexamethasone. Cells were maintained for 8–10 days, with the medium replaced every 48 h. For differentiation of adipocytes from 3T3-L1 cells, the cells were grown until 48 h post-confluence in DMEM+10% FBS. At this time, the medium was replaced with DMEM+3% FBS, 10 µg/mL bovine insulin, 500 µM IBMX, 10 µM troglitazone, 1 µM dexamethasone for three days. After these three days, this medium was replaced with medium supplemented with 3% FBS, 10 µg/mL bovine insulin, and 1 µM dexamethasone for six days, followed by replacement of the medium every 48 h. Insulin, troglitazone, IBMX, and dexamethasone were all purchased from Sigma Aldrich (St. Louis, MO, USA). Human adipose-derived mesenchymal stem cells differentiated into adipocytes via growth in adipocyte differentiation medium and undifferentiated control cells (Lonza Bioscience, Walkersville, MD, USA) were kindly provided by Dr. Beibei Zhu, University of Kentucky (Lexington, KY, USA).

When adenoviral transduction was used in a particular experiment, the adenoviruses were administered 24–48 h after siRNA transfection, and approximately 24 h prior to addition of adipocyte differentiation medium to the cells.

### 2.5. Antibodies

Antibodies for IgG and GAPDH (G9) were from SCBT. Par-4 polyclonal antibody was custom made by Proteintech Biotechnology Company, Rosemont, IL, USA [21]. The PPARγ antibody (C26H12) was from Cell Signaling, Danvers, MA, USA and the β-Actin antibody (AC-74, A5316) was from Sigma Aldrich, St. Louis, MO, USA.

### 2.6. Western Blot Analysis

Cells were lysed using radioimmunoprecipitation assay (RIPA) buffer (Thermo Fisher Scientific Inc., Waltham, MA, USA) combined with Complete Protease Inhibitor Cocktail (Roche; Indianapolis, IN, USA) on ice. Total protein levels were normalized using a Bradford colorimetric assay (BioRad; Hercules, CA, USA), and proteins were denatured by boiling for five minutes in added Laemmeli buffer (Sigma Aldrich, St. Louis, MO, USA). The proteins recovered were resolved using SDS-PAGE and subsequent immunoblotting. Protein band intensity as measured by pixel counts in the gel documentation system (UVP) was obtained using Vision Works LS software (733-2035, VWR; Radnor, PA, USA).

### 2.7. Real-Time qPCR

Total RNA was extracted from MEFs using the RNeasy Plus mini kit (QIAGEN; Valencia, CA, USA). cDNA synthesis was performed using an iScript DNA synthesis kit (Bio-Rad; Hercules, CA, USA). Analysis of gene expression was performed on a 96-well BioRad CFX96 touch real-time thermocycler using iQ SYBR green supermix (BioRad; (Hercules, CA, USA). Samples were run in triplicate and normalized to GAPDH using the ΔΔCT method. Primers were designed using Primer Blast and sourced from Integrated DNA Technologies (IDT; Coralville, IA, USA), as shown in Table 1.

### 2.8. Oil Red O Staining of Cultured Cells

ORO staining was performed as previously described by us [21]. Briefly, conditioned growth medium was removed from the culture wells, and the wells were washed with PBS. Adherent cells were fixed in 10% formalin for 30 min and washed again with PBS. The ORO stain (Sigma Aldrich, St. Louis, MO, USA) was dissolved in isopropanol and water, filtered, and applied to cells for 20 min. At the end of the staining period, the wells were washed with 50% isopropanol, followed with ddH2O wash. The wells were then counterstained with hematoxylin (Sigma Aldrich, St. Louis, MO, USA) for one minute, then washed with ddH2O.

### 2.9. Luciferase Reporter Assays

Luciferase (luc) reporter assays were performed using a Steady State Plus reporter gene assay kit (Perkin Elmer, Waltham, MA, USA). Briefly, cells were co-transfected with the indicated luc reporter constructs and β-Gal expression constructs in a 96-well plate using Lipofectamine and Plus Reagent (Invitrogen; Waltham, MA, USA) according to manufacturer’s protocol. At 24–48 h post-transfection, cell lysates were collected in RIPA buffer containing protease inhibitors (Roche; Indianapolis, IN, USA) for each experimental group, combined with Steady Lite reagent, and analyzed using a Perkin Elmer TopCount plate reader (Perkin Elmer; Waltham, MA, USA), with the signal normalized to β-Gal expression.

### 2.10. Promoter Subcloning

PPARγ2 upstream promoter fragment sequences were amplified from the C57BL/6 mouse genomic DNA template, using primers in Table 2. All forward primers were designed to include a Kpn1 restriction site, and all reverse primers contained an Xho1 restriction site. Amplified fragments were ligated into the multiple-cloning region of a pGL4.14 luciferase reporter vector (Promega; Madison, WI, USA) and amplified in DH5α *E. coli* bacteria. Transformed colonies were selected on ampicillin plates and expanded in LB_amp_ media, and their plasmids were recovered using a maxiprep kit following the manufacturer’s instructions (Origene; Rockville, MD, USA).

### 2.11. Chromatin Immunoprecipitation (ChIP)

ChIP was performed utilizing a ChIP-IT High Sensitivity kit (Active Motif; Carlsbad, CA, USA) according to the manufacturer’s instructions. Briefly, NIH 3T3 cells (~80% confluency) were treated with formaldehyde-containing buffer to fix DNA-binding complexes to the chromatin. These fixed cells were then lysed by repeated snap-freezing cycles and chromatin was sheared via sonication with a Bioruptor Pico device (Diagenode, Denville, NJ, USA)) until the resulting chromatin fragment size was approximately 400–500 bp. Aliquots of recovered sonicated chromatin (15–30 µg) were incubated with 4 µg of relevant antibodies overnight at 4 °C. The antibody-bound chromatin complexes were pulled down using kit-supplied G-protein agarose beads, the crosslinking was reversed, and the DNA eluted and subjected to qPCR. The ChIP-verified primer sets (obtained from Active Motif) are described in Table 3.

### 2.12. Statistical Analysis

All experiments were performed independently at least three different times to verify data reproducibility. The data are expressed as mean ± SEM. Statistical analyses were carried out using either Graphpad prism (version 9.1.1) or Microsoft 365 Excel software, and *p*-values were calculated using Student’s *t*-test.

## 3. Results

### 3.1. Par-4 Inhibits Adipogenesis In Vitro

**Figure 1:**
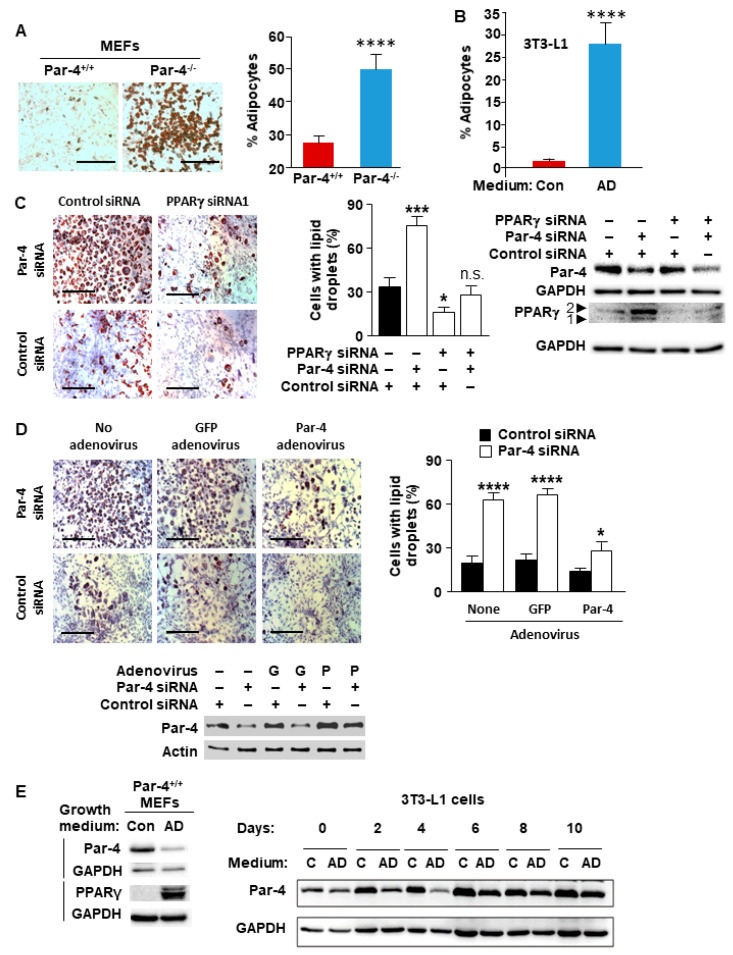
Adipogenesis and PPARγ expression are inversely associated with Par-4 status. (**A**) Loss of Par-4 in MEFs enhances adipogenesis. Par-4^+/+^ and Par-4^−/−^ MEFs were grown in adipocyte differentiation media and subjected to Oil-red O (ORO) staining (left panel). Percentage of ORO-positive cells is shown (right panel). (**B**) Adipogenesis of 3T3-L1 cells was confirmed by growing them in adipocyte differentiation (AD) medium or control (Con) medium and performing ORO staining. Percentage of ORO-positive cells is shown. (**C**) Adipogenesis in 3T3-L1 cells is accelerated by Par-4 knockdown and prevented by PPARγ knockdown. Preadipocyte 3T3-L1 cells were transfected with siRNAs for Par-4 or PPARγ, or co-transfected with these siRNAs, and subjected to treatment with adipogenesis differentiation medium. For control, the cells were treated with scrambled siRNA and maintained in adipogenesis differentiation medium (left panels). After staining the cells with ORO, the percentage of cells with oil droplets was calculated (middle panel). Knockdown of Par-4 and PPARγ was confirmed by Western blot analysis (right panel). (**D**) Adipogenesis in 3T3-L1 cells accelerated by Par-4 knockdown is reversed by Par-4 re-expression. 3T3-L1 cells were transfected with siRNA duplexes for mouse Par-4 or control siRNA and then infected with rat Par-4-expressing adenovirus (P) or control GFP adenovirus (G). The cells were grown in differentiation medium and adipogenesis was examined via oil red O staining (top left panels) and quantified (top right panel). Western blot analysis confirmed Par-4 siRNA knockdown and Par-4 adenoviral expression (bottom panel). (**E**) Par-4 protein expression is downregulated during adipogenesis. Whole-cell extracts were prepared from Par-4^+/+^ and Par-4^−/−^ MEFs (left panel) or 3T3-L1 cells (right panel) grown in normal growth medium (control, C) or in adipocyte differentiation medium (AD) for up to 10 days and subjected to Western blot analysis. (**A**,**C**,**D**) Scale bar, 200 μm. (**A**–**D**) Mean + SEM of three independent experiments shown. Asterisks: (*) indicates *p* < 0.05, (***) indicates *p* < 0.005, and (****) indicates *p* < 0.001; n.s. indicates not significant according to the Student’s *t* test. Molecular weights, β-actin: 42 kDa; Par-4: 40 kDa; GAPDH: 36 kDa; PPARγ: 53,57 kDa.

We used mouse embryonic fibroblasts (MEFs)to determine the role of Par-4 in adipogenesis, as they are pluripotent cells that can differentiate into adipocytes when placed in differentiation medium. Early passage primary MEFs from Par-4^+/+^ and Par-4^−/−^ mice were grown in adipocyte differentiation media as described in the Section 2. After differentiation, the MEFs were stained with oil red O (ORO) and scored for ORO-positive or ORO-negative cells. Par-4 knockout MEFs were more readily differentiated to adipocytes than wild-type MEFs, as demonstrated visually and analytically (Figure 1A). To further validate this observation, we examined the role of Par-4 in the 3T3-L1 pre-adipocyte model for in vitro adipogenesis [31]. As expected, 3T3-L1 pre-adipocytes underwent differentiation to adipocytes, as judged by ORO staining, when they were grown in adipocyte differentiation medium (Figure 1B). We then knocked down Par-4 and/or PPARγ, as a well-recognized master regulator of adipogenesis [32], by transfecting the cells with scrambled control siRNA, mouse Par-4 siRNA, PPARγ siRNA, or co-transfection with Par-4 and PPARγ siRNAs. Par-4 knockdown resulted in a significant increase in the number of ORO-positive cells compared with the control cells (Figure 1C). By contrast, PPARγ knockdown by itself or in combination with Par-4 knockdown was associated with a reduction in the number of ORO-positive cells compared with the control cells (Figure 1C).

### 3.2. Par-4 Overexpression Inhibits Adipogenesis

As Par-4 knockout or knockdown enhances adipogenesis in cell cultures, we decided to investigate whether this effect would be reversed by re-expression of Par-4. In these experiments, transfection of 3T3-L1 cells with Par-4 siRNA reduced Par-4 expression and significantly enhanced adipogenesis relative to cells transfected with control siRNA (Figure 1D). Transduction of these cells with a control GFP adenovirus did not further alter the ORO staining and adipogenesis in control siRNA or siPar-4-transfected cells (Figure 1D). On the other hand, transduction of the cells that were transfected with control siRNA or Par-4 siRNA with Par-4-producing adenovirus resulted in abrogation of adipogenesis (Figure 1D).

### 3.3. Adipogenesis Downregulates Par-4 Expression

In the course of the adipogenesis experiments, we tested the effect of adipogenesis on Par-4 protein expression. We compared Par-4 protein levels in wild-type MEFs as well as in 3T3-L1 cells, which were maintained in adipocyte differentiation medium or control medium. These experiments indicated that the differentiated cells consistently exhibited downregulation of Par-4 expression manifested early during the course of adipogenesis (Figure 1E). As expected, PPARγ expression was also elevated in the differentiated cells (Figure 1E). These findings indicate that the adipogenic pathway rapidly downregulates Par-4 expression.

### 3.4. PPARγ Expression Is Inversely Associated with Par-4 Status

**Figure 2:**
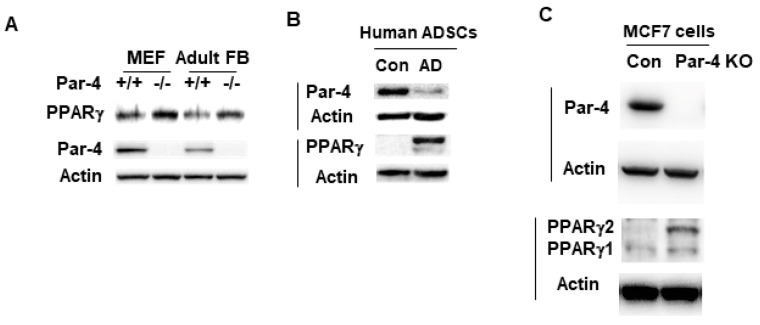
PPARγ expression is inversely associated with Par-4 expression. MEFs or adult fibroblasts from Par-4^+/+^ and Par-4^−/−^ mice (**A**), human adipose-derived stem cells (ADSCs) differentiated into adipocytes by growing them in adipocyte differentiation (AD) medium or undifferentiated control cells (Con) (**B**), or MCF7 cells with CRISPR/Cas9 induced Par-4 knockout (Par-4 KO) or control cells (**C**) were lysed in RIPA buffer and the whole-cell lysates were subjected to Western blotting for Par-4, actin, and PPARγ.

As PPARγ is a key regulator of adipogenesis, we screened the cell lysates from Par-4^+/+^ and Par-4^−/−^ MEFs, as well as Par-4^+/+^ and Par-4^−/−^ adult mouse lung primary fibroblasts, for expression of PPARγ. As seen in Figure 2A, PPARγ was found to be upregulated in Par-4^−/−^ MEFs relative to Par-4^+/+^ MEFs. PPARγ was upregulated in adult fibroblasts isolated from Par-4^−/−^ mice relative to those isolated from Par-4^+/+^ mice. To confirm that these findings were not solely applicable to mouse cells, this experiment was repeated using lysates from human cells. Human adipose-derived stem cells that differentiated to adipocytes in adipocyte differentiation medium showed reduced levels of Par-4 and elevated levels of PPARγ relative to undifferentiated control cells (Figure 2B). Moreover, human MCF7 cells in which Par-4 expression was knocked out demonstrated PPARγ upregulation relative to the corresponding control cells (Figure 2C). Collectively, these experiments indicate that loss of Par-4 is consistently associated with upregulation of PPARγ expression in mouse and human cells.

### 3.5. Par-4 Transcriptionally Inhibits PPARγ

**Figure 3:**
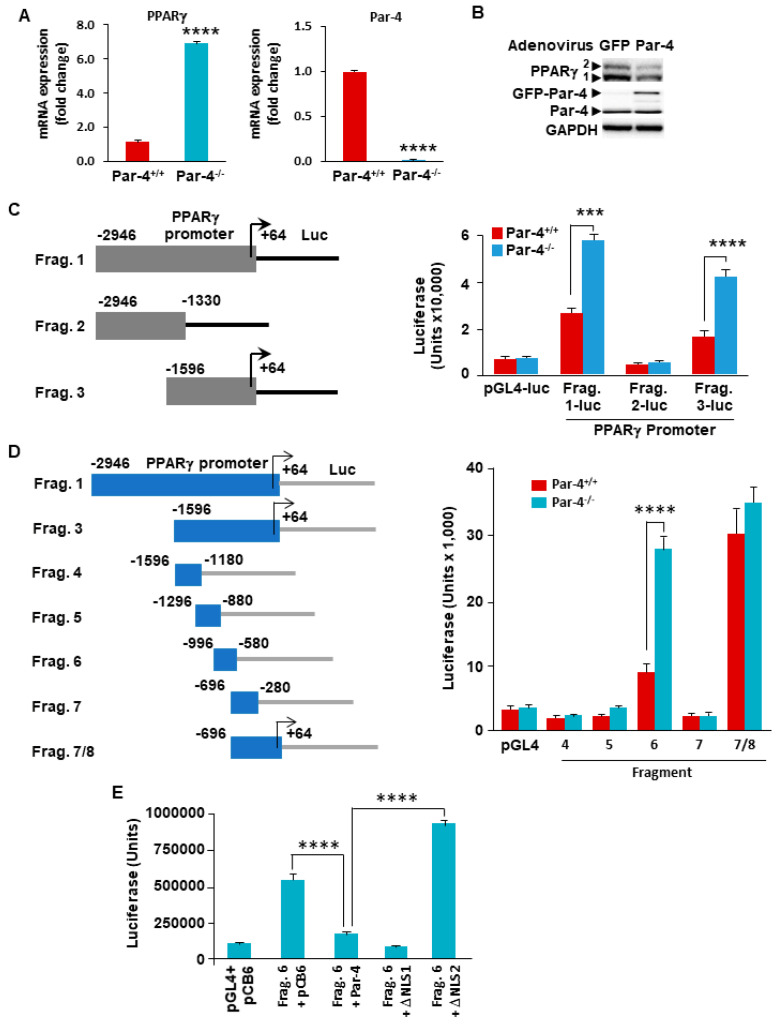
PPARγ gene transcription is inversely associated with Par-4 expression. (**A**) Par-4^−/−^ MEFs display increased transcription of PPARγ. RNA was extracted from Par-4^+/+^ and Par-4^−/−^ MEFs and subjected to qPCR for Par-4, PPARγ, and GAPDH. Data normalized to corresponding GAPDH levels are shown. (**B**) PPARγ expression is inhibited by Par-4 overexpression. 3T3-L1 cells were infected with GFP or GFP-Par-4 producing adenovirus, and whole-cell lysates were subjected to Western blot analysis. (**C**) Generation of luciferase constructs containing PPARγ2 promoter deletion fragments 1, 2, and 3. The deletion fragments 1, 2, and 3 of the mouse PPARγ (isoform 2) promoter were cloned into pGL4 luciferase expression constructs (left panel). MEFs were transfected with either the luc constructs containing PPARγ promoter fragments or an empty pGL4, in the presence of a β-galactosidase (β-gal) expression construct. Whole-cell extracts were then subjected to luciferase activity assays. The luciferase activity normalized to β-gal activity is shown for Fragments (Frag) 1, 2, and 3 (right panel). (**D**) Deletion fragment 6 is necessary for Par-4-mediated regulation of the PPARγ2 promoter. PPARγ promoter Fragment 3 was subdivided into five smaller fragments (left panel), and the luc assay was repeated as above in MEFs. Luciferase activity normalized to β-gal is shown (right panel). (**E**) Nuclear entry is necessary for Par-4 mediated regulation of the PPARγ2 promoter. Par-4^−/−^ MEFs were co-transfected with the luc construct containing fragment 6 along with a β-gal expression vector combined with (i) an empty pCB6 control plasmid, (ii) full length Par-4-expression plasmid, (iii) Par-4 plasmid containing deletion of NLS1 sequence (ΔNLS1), or (iv) Par-4 plasmid with deletion of both NLS1 and NLS2 (ΔNLS2). The whole-cell lysates were subjected to luciferase assays; luciferase activity normalized to β-gal is shown. (**A**,**C**–**E**) Means of 3 experiments + SEM are shown. Asterisks: (***) indicates *p* < 0.005 and (****) indicates *p* < 0.001 according to the Student’s *t* test.

As the expression of PPARγ is upregulated under conditions of Par-4 knockout or downregulation, and as Par-4 is known to act as a transcriptional repressor, we tested whether PPARγ was regulated by Par-4 transcriptional activity as previously noted for other genes [15,16]. We used real-time qPCR of cDNA synthesized from the RNA of Par-4^+/+^ and Par-4^−/−^ MEFs, utilizing primers for Par-4, PPARγ, and GAPDH. After normalizing the qPCR data for GAPDH, we noted significant transcriptional upregulation of PPARγ in the Par-4^−/−^ MEFs (Figure 3A).

### 3.6. Par-4 Overexpression Inhibits PPARγ1 and γ2 Isoforms

As PPARγ is upregulated under Par-4 ablation, we tested whether Par-4 overexpression could suppress PPARγ expression. Par-4 was overexpressed in 3T3-L1 cells by transducing the cells with Par-4-producing adenovirus, using the GFP-producing adenovirus as control. Whole-cell lysates were obtained from transduced cells and subjected to Western blot analysis for PPARγ, Par-4, and GAPDH. These experiments indicated that the fibroblasts infected with the Par-4 adenovirus expressed significantly lower levels of PPARγ1 and PPARγ2 protein isoforms relative to the cells infected with GFP-adenovirus (Figure 3B).

### 3.7. Par-4 Inhibits Transcriptional Activity of the PPARγ2 Promoter

As the expression of PPARγ was repressed by Par-4, it was apparent that Par-4 was functioning as a transcriptional repressor. The LZ domain of Par-4 lacks flanking basic amino acid regions for direct DNA binding; therefore, Par-4 is not expected to directly interact with the DNA. However, Par-4 can bind other DNA-binding proteins that bring it to the DNA to serve as a transcriptional repressor. To address this question, we tested whether Par-4 affected the activity of the PPARγ2 promoter, as the PPARγ2 isoform is considered a key regulator of adipogenesis [30]. We first generated a series of luciferase (luc) constructs of the upstream promoter for PPARγ2, using sequences from the 3 kb region upstream of the start codon (ATG) (Figure 3). Our initial luc constructs included the entire 3 kb upstream region (designated Fragment 1/Frag. 1), the distal 1.6 kb region (Frag. 2), and the 1.6 kb region proximal to the ATG site (Frag. 3) (Figure 3C). The constructs were co-transfected along with a β-Gal expression construct into Par-4^+/+^ and Par-4^−/−^ MEFs and tested by luciferase expression assays, normalized to β-Gal activity. These experiments indicated that luciferase expression was enhanced in Par-4^−/−^ MEFs transfected with Frag. 1 and Frag. 3 (Figure 3C). By contrast, the luciferase activity of Frag. 2 was comparable to that of the empty vector control, regardless of Par-4 status (Figure 3C). These findings indicate that Par-4 regulates the expression of the PPARγ2 promoter region within 1.6 kb of the PPARγ2 start codon.

To further map the PPARγ2 promoter region regulated by Par-4, we generated additional luc constructs by subcloning parts of the Frag. 3 in the luc-reporter vector (Figure 3D). These fragments were about 450 base pairs (bp) in length and overlapped the adjacent fragment sequences by approximately 100 bp. Luc assay in Par-4^+/+^ and Par-4^−/−^ MEFs indicated that the Par-4^−/−^ MEFs transfected with the construct containing Frag. 6 displayed significantly increased activity compared with the control cells. No significant difference in luc activity was observed in Par-4^+/+^ and Par-4^−/−^ MEFs for the other fragments tested (Figure 3D). These findings indicate that Par-4 acts upon a sequence between 580 bp and 996 bp upstream of the transcription initiation site of the PPARγ2 promoter (Figure 3D).

### 3.8. Nuclear Localization of Par-4 Is Required for Regulation of the PPARγ2 Promoter

As Par-4 is localized in multiple cellular compartments, we investigated whether nuclear localization of Par-4 is required for its effects on the PPARγ2 promoter. We made use of PGL3-based constructs containing either a complete cDNA clone of Par-4 or deletion mutants of Par-4 from which the nuclear localization sequences were deleted (designated ΔNLS1 or ΔNLS2). Each of these constructs or an empty control vector was co-transfected with either Frag. 6 or an empty luc vector into Par-4^−/−^ MEFs. The cells were also co-transfected with a β-Gal construct for normalization. Our intent was to compare the effects of full-length Par-4 with those seen for ΔNLS1 or ΔNLS2 with regards to Frag. 6 Luc activity. These luc reporter assays revealed that co-transfection with the full-length Par-4 construct or the ΔNLS1 mutant significantly inhibited the luc activity from Frag. 6. However, co-transfection with the ΔNLS2 mutant completely abrogated the effects of Par-4 (Figure 3E). As Par-4 and ΔNLS1 but not ΔNLS2 can translocate to the nucleus, these findings indicate that nuclear localization of Par-4 is required for Par-4-mediated downregulation of PPARγ2.

### 3.9. Par-4 Binds the PPARγ2 Promoter

**Figure 4:**
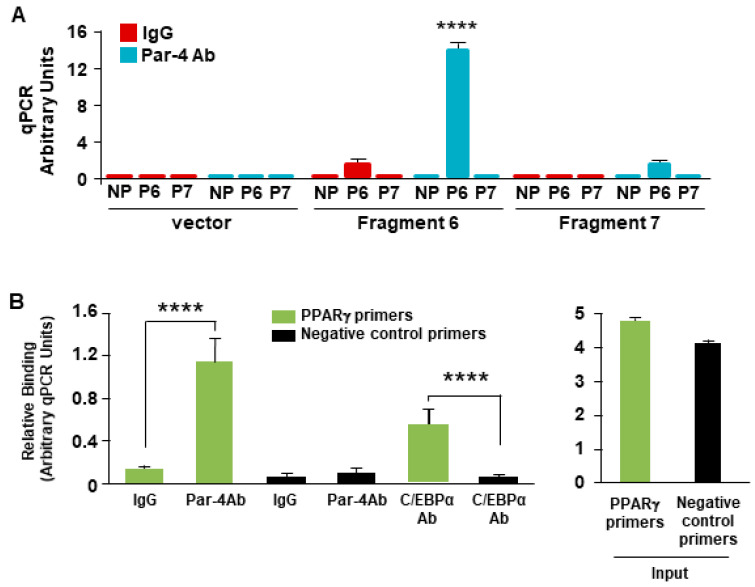
Par-4 binds to the PPARγ promoter. (**A**) Endogenous Par-4 protein binds the PPARγ2 promoter sequence in Fragment 6. NIH 3T3 cells were transfected with either an empty control vector, Fragment 6-containing plasmid, or Fragment 7-containing plasmid. These transfected cells were then subjected to ChIP with pull-down accomplished with either anti-Par-4 antibody (Ab) or IgG control Ab. Immunoprecipitated DNA fragments were analyzed using primers for Fragment 6, Fragment 7, or negative control primers. (**B**) Endogenous Par-4 protein binds the endogenous PPARγ2 promoter region. Non-transfected NIH 3T3 cells were subjected to ChIP analysis with either the anti-Par-4 antibody (Ab), IgG control Ab or C/EBPα Ab. Immunoprecipitated DNA fragments were analyzed using primers for Fragment 6, C/EBP positive-control primers, or negative control primers. (**A**,**B**) Means of 3 experiments + SEM are shown. Asterisk (****) indicates *p* < 0.001 according to the Student’s *t* test.

As the nuclear entry of Par-4 is a requisite for transcriptional repression of the PPARγ2 promoter, we determined whether Par-4 exhibited chromatin binding as a component of a co-repressor complex in the region. We utilized chromatin immunoprecipitation (ChIP) analysis to determine whether an anti-Par-4 antibody could pull down the genomic sequence region corresponding to Frag. 6. NIH 3T3 cells were transfected with either a pGL4.14 control vector, a Frag. 6-containing construct or a Frag. 7-containing construct (as a negative control). The fragmented chromatin from all transfection groups was subjected to pull-down with either a Par-4 antibody or IgG control antibody. The resulting ChIP DNA from all groups was subjected to qPCR, utilizing primers specific to Frag. 6, Frag. 7, or a negative control primer, designated P6, P7, and NP, respectively. After the qPCR data were normalized to GAPDH in the input DNA for all groups, we noted that the anti-Par-4 antibody pulled down Frag. 6 but not Frag 7, while the IgG control antibody did not pull down either fragment (Figure 4A).

We next tested whether Par-4 could pull down the endogenous PPARγ2 promoter sequence of interest in Frag. 6. We performed ChIP studies on NIH 3T3 cells with antibodies for Par-4, IgG, and C/EBPα. The C/EBPα antibody was used as a positive control because Frag. 6 contains a putative C/EBPα binding site [33]. qPCR results showed significant amplification of the Frag. 6 sequence in the Par-4 and C/EBPα antibody pull-down groups compared with the IgG antibody pull-down group (Figure 4B). These findings indicate that Par-4 binds to a chromatin element within a specific region of Frag. 6 in the endogenous PPARγ2 promoter.

## 4. Discussion

Par-4 promotes obesity in chow-fed mice, yet the precise mechanism by which Par-4 regulates adipogenesis to sustain the increased requirement for fat storage in Par-4-null mice had not been fully delineated. Cell culture studies provide user-friendly and often relevant models to determine the mechanism underlying biological processes. We therefore used MEFs that were pluripotent and known to differentiate into mature adipocytes, as well as 3T3-L1 pre-adipocytes that differentiated into mature adipocytes when placed in differentiation medium and recapitulated the process of adipogenesis occurring in vivo [34,35]. Our present studies indicate that MEFs from Par-4 conventional (whole body) knockout mice readily undergo differentiation to adipocytes with an increase in lipid droplet accumulation relative to MEFs from wild-type mice. Moreover, knockdown of Par-4 in 3T3-L1 pre-adipocyte cultures promoted their differentiation to mature, lipid-loaded adipocytes. These studies indicate that Par-4 suppresses adipogenesis and lipid accumulation. Consistent with these observations, Par-4 replenishment prevented lipid accumulation and adipogenesis. This action of Par-4 was associated with regulation of PPARγ2, which is known to induce adipogenesis. Par-4 loss increased PPARγ2 expression and, contrariwise, Par-4 overexpression inhibited PPARγ2 expression. Par-4 was found to bind to the PPARγ2 promoter to regulate its expression at the transcription level. PPARγ is a master regulator of adipocyte differentiation, and our studies indicated that Par-4 regulates both isoforms of PPARγ and particularly PPARγ2 transcription by suppression of the PPARγ2 promoter to regulate adipogenesis. Thus, PPARγ is a novel target of Par-4 that may play a role in adipogenesis associated with obesity regulation by Par-4.

Intracellular Par-4 found in the nucleus is not known to directly bind to the DNA but serves as a transcriptional repressor when brought to the DNA by another DNA-binding protein [15,16]. Interestingly, our ChIP and luciferase-reporter experiments indicated that Par-4 bound to the promoter of PPARγ2 and suppressed its transcription. The upstream region of PPARγ2 binding to Par-4 was localized to an approximately 400 bp promoter fragment. As the JASPAR database indicates that a number of transcription factors are expected to bind to this PPARγ2 promoter region, future in-depth studies may elucidate the precise binding site(s) and the transcription factor(s) associated with this Par-4 binding site.

Par-4 loss promotes obesity in mice by upregulating the p53/21 pathway, as well as LPL in the adipose tissue [21]. LPL is known to be expressed on the surface of endothelial cells lining the vasculature in adipose tissues. Thus, multiple cell types in the adipose tissue may influence the process of adipogenesis and contribute to obesity in live rodents or humans. Increased fat accumulation in the adipocytes of Par-4 knockout mice was accommodated by hypertrophic enlargement of the adipocytes [21]. Adipocyte hypertrophy was consistent with insulin resistance in the Par-4 knockout obese mice [21]. The present cell culture studies indicated an increase in mature adipocytes upon loss of Par-4, implying a primary role for Par-4 in adipogenesis. These cell culture findings are consistent with the robust increase in PPARγ2 expression, which is known to be associated with adipogenesis. However, PPARγ is not significantly induced in the adipose tissue of Par-4 knockout mice that are obese [21]. It is plausible that the effect of Par-4 loss on upregulation of PPARγ, which could theoretically lead to adipocyte hyperplasia and insulin sensitivity [36], is masked in Par-4 knockout mice by the dominant hypertrophic gene program induced directly or indirectly by Par-4 loss in mouse adipose tissues that consist of multiple cell types within the adipocyte microenvironment.

In summary, Par-4 loss is associated with adipogenesis in pluripotent cells as well as in pre-adipocytes in cell culture models. Regulation of adipogenesis by Par-4 is attributed to suppression of PPARγ2 expression by Par-4 binding to its proximal promoter region. Thus, Par-4 suppresses the basic process of adipogenesis by repression of the master regulator PPARγ. As an inverse relationship between Par-4 and PPARγ was noted across mouse embryonic cells, pre-adipocytes, and adult fibroblasts, regulation of PPARγ by Par-4 can be further explored using advanced human adipose tissue culture methods [37], as well as obesity and diabetes models [38,39], to restore insulin sensitivity. Moreover, in view of the tumor suppressor function of Par-4 [40,41,42,43,44,45,46,47,48,49,50,51,52], its inverse relationship with PPARγ, and abnormal lipid metabolism caused by increased PPARγ expression in cancer [53,54,55,56,57], it will be important to determine the functional significance of the inverse relationship between Par-4 and PPARγ in cancer.

## 5. Conclusions

In conclusion, Par-4 suppresses PPARγ2 expression by binding to its proximal promoter region. Adipogenesis in pluripotent cells, as well as in pre-adipocyte cell cultures is accelerated by Par-4 loss that results in induction of the master regulator PPARγ.

## Figures and Tables

**Table 1:** Primers for real-time qPCR.

Target	Forward Primer (5′-3′)	Reverse Primer (5′-3′)
Mouse Par-4	AGAATGAAGCTGCGACCCTC	ATCTTCTGGGGCACTGGTTG
Mouse PPARγ1	GTCTCGGTTGAGGGGAC	TGTCAACCATGGTAATTTCAGT
Mouse GAPDH	AAATGGTGAAGGTCGGTGTG	TGAATTTGCCGTGAGTGGAG

**Table 2:** Primers for promoter subcloning.

Fragment	Forward Primer (5′-3′)	Reverse Primer (5′-3′)
1	GAGTGGTACCGTAAGCAACATTTATTG	GAGCCTCGAGAACAGCATAAAACAGAG
2	GAGTGGTACCGTAAGCAACATTTATTG	GAGCCTCGAGTTTAACAAGAATTCTTA
3	GAGTGGTACCTTTTACATTCTAGACAC	GAGCCTCGAGAACAGCATAAAACAGAG
4	GAGTGGTACCTTTTACATTCTAGACAC	GAGCCTCGAGGGTCTAAATATCAGTCA
5	GAGTGGTACCCATCATTTGGACTACTG	GAGCCTCGAGGCCTTTGCCCTTTTTGG
6	GAGTGGTACCGCTCTTTTAAAGTCCAC	GAGCCTCGAGAGGTCCAAAATGTTACT
7	GAGTGGTACCGATAGATAAACAAATTT	GAGCCTCGAGGTACAGTAGTTGGAATT
7 + 8	GAGTGGTACCGATAGATAAACAAATTT	GAGCCTCGAGAACAGCATAAAACAGAG

**Table 3:** ChIP primers.

Primer Pair Designation	Forward Primer (5′-3′)	Reverse Primer (5′-3′)
6	GCTCTTTTAAAGTCCACAAGTCACTG	GGAAAACTCTGGCTTCTTGCTTAA
7	ATGTGTGATTAGGAGTTTCAACCAAA	GAATTACCAGAGCAGAGATTGTTCA
Mouse Negative Control Primer Set 2	Proprietary Sequence	Proprietary Sequence
Mouse Positive Control Primer Set (GAPDH)	Proprietary Sequence	Proprietary Sequence

## Data Availability

The original contributions presented in the study are included in the article, further inquiries can be directed to the corresponding author.
